# Efficient CRISPR-Cas genome editing in brown algae

**DOI:** 10.1016/j.crmeth.2025.101273

**Published:** 2025-12-30

**Authors:** Cláudia Martinho, Masakazu Hoshino, Morgane Raphalen, Viktoriia Bukhanets, Anagha Kerur, Kenny A. Bogaert, Rémy Luthringer, Susana M. Coelho

**Affiliations:** 1 Department of Algal Development and Evolution, Max Planck Institute for Biology, Max-Planck-Ring 5, 72076 Tübingen, Germany; 2 School of Life Sciences, University of Dundee, Division of Plant Sciences at The James Hutton Institute, Errol Road, DD2 5DA Dundee, UK; 3 Research Center for Inland Seas, Kobe University, Rokkodai 1-1, Nadaku, Kobe 657-8501, Japan Kobe

**Keywords:** brown algae, CRISPR-Cas, genome editing, RNP transfection

## Abstract

Brown algae represent the third most complex lineage to have independently evolved multicellularity, distinct from plants and animals. Yet, functional studies of their development and evolution have been limited by the absence of efficient genome editing tools. Here, we present a robust, high-efficiency, and transgene-free CRISPR-based genome editing platform applicable across four ecologically and biotechnologically important brown algal species. Using *Ectocarpus* as a model, we optimized a polyethylene glycol (PEG)-mediated ribonucleoprotein (RNP) delivery system that achieves reproducible editing across multiple loci without cloning or specialized equipment. As proof of concept, we recreated the hallmark *imm* mutant phenotype by precisely editing the *IMMEDIATE UPRIGHT* (*IMM*) locus. APT/2-fluoroadenine (2-FA) selection further enhanced specificity with minimal false positives. The method was easily transferable to other species, including kelps. This platform now enables functional genomics in brown algae, providing powerful tools for investigating development, life cycle regulation, and the independent evolution of complex multicellularity.

## Highlights

• We develop high-efficiency, transgene-free CRISPR genome editing for brown algae
• We optimize PEG-RNP delivery to achieve reproducible editing across multiple loci
• We demonstrate precise editing of the *imm* locus in *Ectocarpus*
• We provide a platform to enable functional genomics in brown algae

## Motivation

Although most of the biodiversity on Earth lives in oceans, a significant proportion of its organisms remain largely uncharacterized. Brown algae represent one such understudied group of marine photosynthetic eukaryotes. Despite their importance as emerging models for developmental evolution and blue biotechnology, functional genomics in brown algae has remained largely inaccessible due to a lack of efficient and scalable genome editing tools. Our aim is to democratize genome editing in brown algae by developing a high-efficiency, transgene-free protocol that works across multiple species, without the need for specialized equipment.

## Graphical abstract

**Figure:**
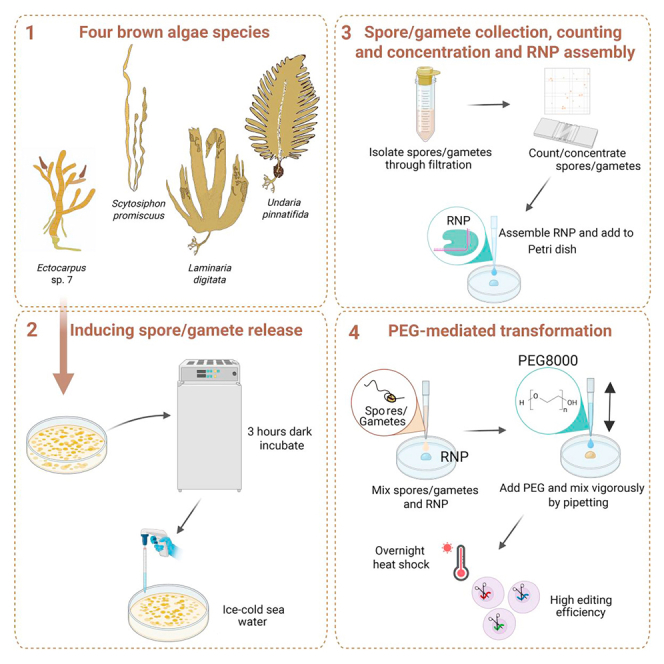

## Teaser

Brown algae, a key yet understudied lineage of multicellular eukaryotes, have long lacked tools for functional genomics. Martinho et al. introduce a high-efficiency, transgene-free CRISPR platform enabling precise genome editing across multiple brown algal species, unlocking new possibilities for studying their development, evolution, and biotechnological potential.

## Introduction

Brown algae represent a lineage of marine photosynthetic eukaryotes within the stramenopiles (heterokonts) that evolved complex multicellularity independently of plants and animals^1^ through mechanisms that remain largely unexplored.^2^^,^^3^^,^^4^^,^^5^ Ubiquitous in coastal environments, they play key ecological roles, serve as a natural source of bioactive compounds for industrial applications, and constitute an important food resource.^6^ However, the lack of efficient and broadly accessible genome editing protocols has hindered both fundamental and applied research.

Genome editing protocols using recombination-based methods and DNA-free ribonucleoprotein (RNP)-based approaches have been successfully established in unicellular stramenopiles such as diatoms. Delivery methods in diatoms range from biolistics to electroporation and yield high genome editing efficiency (reviewed in Li et al.^7^). Methods to transform macroalgae outside the stramenopiles have also been described, including in the red algae *Gracilariopsis lemaneiformis* and *Neopyropia yezoensis*, which also employ biolistics to deliver RNPs or plasmid DNA to thallus tissue.^8^^,^^9^ However, note that, in these species, this approach results in the generation of chimeric tissues containing gene knockout (KO) mutants and wild-type (WT) cells.^8^^,^^9^ In the multicellular green alga *Ulva* spp., gene KOs can be generated with high efficiency via RNPs or plasmid DNA gamete delivery with polyethylene glycol (PEG).^10^^,^^11^ Within multicellular stramenopiles, the brown algae *Ectocarpus* sp. 7 and *Saccharina japonica* were successfully edited by employing biolistic and/or microinjection.^12^^,^^13^ However, these labor-intensive approaches rely on specialized equipment, require extensive technical training, and are limited by relatively low editing efficiencies; for instance, biolistic delivery in *Ectocarpus* sp. 7 has been associated with a false-positive rate of approximately 50%.^12^

Here, we develop a PEG-mediated RNP transfection method, previously shown to enable highly efficient genome editing in the green alga *Ulva prolifera*, for use in brown algae. This approach achieves unprecedented efficiency with negligible false-positive rate across four brown algae: *Ectocarpus* sp. 7 and *Scytosiphon promiscuus*, widely used systems for fundamental research^14^^,^^15^^,^^16^^,^^17^; *Laminaria digitata*, a laminariacean kelp of ecological and economic importance; and *Undaria pinnatifida* (wakame), an alariacean kelp and one of the most commercially cultivated brown algae worldwide.^6^

## Results

### PEG-mediated RNP *Ectocarpus* gamete transfection allows highly efficient genome editing

**Figure 1:**
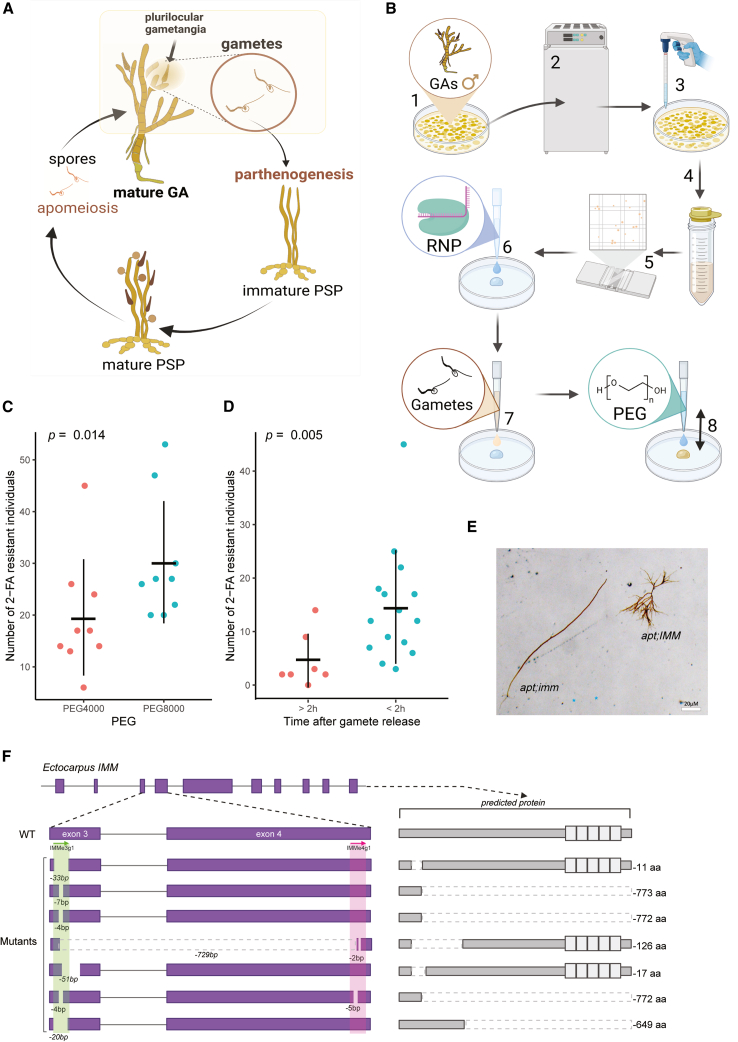
*Ectocarpus* CRISPR-based genome editing (A) Parthenogenetic life cycle of *Ectocarpus* sp. Gametophyte (GA) produces gametes that parthenogenetically develop into parthenosporophyte (PSP). (B) Schematic diagram of the process of the PEG transfection for *Ectocarpus sp*. 7. The process begins with GAs cultured in Petri dishes (step 1), followed by incubation to induce gamete release (step 2). The gametophytes are then transferred using a pipette (step 3), filtered to separate from gametophytic tissue, and centrifuged to concentrate gametes (step 4). After centrifugation, the concentration of gametes is examined using a hemocytometer (step 5). Ribonucleoprotein (RNP) is added to the Petri dish (step 6) and mixed with gamete suspension (step 7). Finally, 40% w/v PEG, prepared in seawater, is applied to facilitate transfection and vigorously mixed by pipetting with a wide bore tip (step 8). See details in STAR Methods section. (C) Comparison of the number of 2-FA-resistant individuals following treatment with different PEG molecular weights: PEG4000 and PEG8000. Dots depict independent trials (Table S2). The horizontal and vertical lines in the scatterplot represent the mean and standard deviation, respectively. Significantly more resistant individuals were observed with PEG8000 treatment (exact Wilcoxon rank-sum test, *p* = 0.014). (D) Comparison between PEG treatments using gametes collected more than 2 h after release (>2 h) and those using freshly released gametes (<2 h). A significantly higher number of resistant individuals was observed when fresh gametes were used (exact Wilcoxon rank-sum test, *p* = 0.005). (E) Morphological difference of double KO mutant of *APT* and *IMM* (*apt;imm*) and *APT* single KO mutant (*apt;IMM*). The *APT* single KO mutant develops prostrate filaments, whereas the double KO mutant develops long upright filaments. (F) Schematic representations of the *Ectocarpus IMM* gene model of WT and mutant individuals. Exon regions are shown as purple boxes, and crRNAs are indicated with arrows. The expected protein products for the WT and each mutant are shown to the right of their respective gene models. The five imperfect tandem repeats of a 38 amino acid cysteine-rich motif, characteristic of the IMM C-terminal region, are represented as white boxes. See also Tables S1 and S2.

To develop a simple and highly efficient genome editing method in the model alga *Ectocarpus* sp., we exploited its ability to regenerate haploid parthenosporophytes (PSPs) from unfertilized gametes via parthenogenesis (Figure 1A). *Ectocarpus* gametes remain cell wall-free for approximately 2 h following release from gametangia in the absence of fertilization,^18^ providing a transient window during which CRISPR-Cas-RNPs can be efficiently transfected.

To transfect Cas-RNPs, we employed PEG-mediated RNP transfection, previously successful in the green alga *U. prolifera*.^11^ Though mature male *Ectocarpus* gametophytes (GAs) release abundant swimming gametes^19^^,^^20^ (Figure 1A) and were therefore used to optimize the protocol, mutations can be easily backcrossed into WT females,^19^^,^^20^ provided that fertility is not affected. Because *Ectocarpus* is cultivated at relatively low temperatures (14°C), we used an engineered Cas12a variant, LbCas12a (IDT), which performs efficiently across a wide range of temperatures.^21^ We isolated male gametes from cultivated algae and transfected them with LbCas12a-RNPs using 40% w/v PEG (Figure 1B, see STAR Methods). As a selection marker, we used LbCas12a loaded with an *APT* locus CRISPR RNA CRISPR RNA (crRNA) (*Ec-28_000520*, Table S1) to generate apt loss-of-function mutations, which enable survival in selective medium supplemented with 2-fluoroadenine (2-FA).^12^

Given that heat stress increases CRISPR-induced mutagenesis efficiency in land plants,^22^^,^^23^ we tested the effect of an overnight heat shock at 22°C in darkness after PEG transfection (Figure S1A) and varied the LbCas12a-to-crRNA ratios (Figure S1B), with no statistically significant effect on the number of 2-FA resistant obtained (Figures S1A and S1B; Table S2A). However, the heat-shock step was maintained since it resulted in a higher number of 2-FA-resistant individuals (Figure S1A). Additionally, we tested the effect of different PEG molecular weights and observed that PEG8000 resulted in a higher number of 2-FA-resistant individuals (0.003% of transfection efficiency) averaging 30.3 2-FA-resistant individuals per trial i.e., per Petri dish (Figure 1C; Table S2A). Data analysis from multiple independent experiments during protocol optimization revealed that transfection within the first 2 h after gamete release significantly improves editing efficiency (Figure 1D; Table S2A), and thus, it is recommended to proceed with transfection as soon as gamete release is completed.

Given the method’s high efficiency in generating 2-FA-resistant individuals, we next tested whether it could produce double mutants targeting both *APT* and a gene of interest. We therefore designed two LbCas12a crRNAs targeting exons 3 and 4 of the *IMMEDIATE UPRIGHT* (*IMM*, *Ec-27_002610.1*) gene and co-transfected them with the *APT* RNP complex (Table S1). Mutations in the *IMM* locus are known to disrupt basal cell development and accelerate upright filament formation in *Ectocarpus* sp. providing a clear developmental phenotype to assess the efficiency of our method (Figure 1E). Remarkably, we observed this characteristic *imm* phenotype in 19 individuals out of 59 (32%) of 2-FA-resistant individuals following co-transfection. We selected 15 individuals displaying the *imm* phenotype and confirmed, employing Sanger sequencing, that all harbored mutations at both the *APT* and *IMM* loci (Table S2B). In contrast, three 2-FA-resistant individuals with a WT phenotype showed mutations only at the *APT* locus (Table S2B). Notably, one of the 15 double mutants carried a 723 bp deletion between the two *IMM* target sites, while others had smaller indels at individual sites (Figure 1F; Table S2B). Although crRNA targeting exon 3 produced mutations in all tested individuals, crRNA targeting exon 4 generated mutations in only 2, suggesting that crRNA or target site susceptibility may differ. Importantly, no false positives were observed since all algae that grew on selective medium and tested carried mutations in *APT*, confirming the high precision and efficiency of the co-transfection protocol.

### PEG-mediated RNP transfection results in high-frequency genome edits across multiple brown algal species

**Figure 2:**
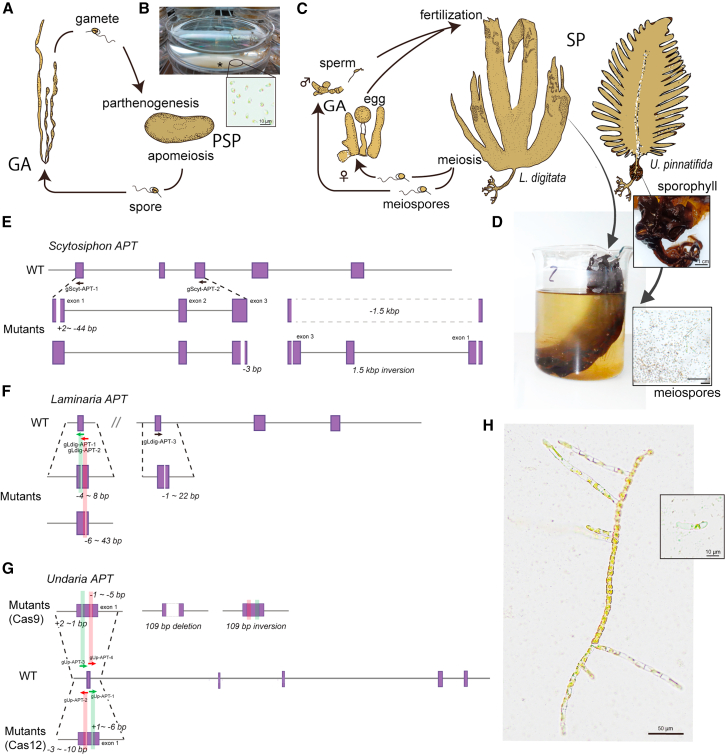
*Scytosiphon* and kelp CRISPR-based genome editing (A) Parthenogenetic life cycle of *Scytosiphon promiscuus*. Macroscopic gametophytes (GAs) alternate with microscopic discoidal parthenosporophyte (PSP). The figure is based on Hoshino.^24^ (B) Suspension of male gametes of *S*. *promiscuus* showing accumulation of gametes (asterisk) by negative phototaxis. (C) Life cycle of *Laminaria digitata* and *Undaria pinnatifida*. In both species, microscopic GAs alternate with macroscopic sporophyte (SP; left, *Laminaria* sporophyte; right, *Undaria* sporophyte). (D) Released meiospores from *Laminaria* sori and *Undaria* sporophylls. (E) Schematic representations of the *S. promiscuus APT* gene model of WT and mutant individuals. See details in Figure 1. (F) Schematic representations of the *L. digitata APT* gene model of WT and mutant individuals. (G) Schematic representations of the *U. pinnatifida APT* gene model of WT and mutant individuals following Cas9 transformation (above) and LbCas12a transformation (below). (H) One-month-old germlings of *S. promiscuus APT* mutant and WT (right top) in selective medium. See also Tables S1 and S2.

To test the transferability of the PEG-mediated RNP transfection protocol beyond *Ectocarpus*, we applied the method to other non-model brown algae. Specifically, we transfected *APT*-targeting RNPs into *S. promiscuus* and the kelps *L. digitata* and *U. pinnatifida* (Figure 2), introducing only minor protocol modifications. For instance, in *S. promiscuus* and *L. digitata*, effective genome editing was achieved even when the amount of LbCas12a was reduced to less than half of that used in the PEG-mediated protocol in *Ectocarpus* (125–50 pmol LbCas12a; see STAR Methods). In *S. promiscuus*, as in *Ectocarpus*, gametes released from laboratory-cultivated algae were used for PEG transfection (Figures 2A and 2B). For the kelps, we instead used meiospore transformation (Figures 2C and 2D), since kelp gametes exhibit limited parthenogenetic capacity. Meiospores develop into haploid gametophytes, allowing direct phenotypic evaluation of mutation effects without the need for backcrossing or generating homozygous lines.

The transfection rate in *S. promiscuus* was approximately 0.25%, allowing to retrieve between 107 and 2,458 2-FA-resistant individuals (Table S2C) and suggesting that a lower number of input cells per transformation could potentially be used and still yield a significant number of transformants. In *L. digitata*, transformation efficiency varied between 0% and 0.79% and yielded between 0 and 6,128 2-FA-resistant individuals, depending on the trial (Table S2C). This discrepancy was likely due to the lack of fresh zoospores—i.e., actively swimming, wall-less cells—in trials 1 and 3. Instead, non-motile spores, possibly bearing cell walls, were used, and the presence of the cell wall likely reduced the efficiency of construct delivery (Table S2C). In *U. pinnatifida*, the transfection efficiency among trials was between 0.014% and 0.021%, reflecting more than 100 2-FA-resistant individuals per trial. In *L. digitata*, genotyped 2-FA-resistant individuals exhibited mutations in the *APT* locus with a false-positive rate of 11.9% ± 19.2% among the trials (mean ± SD; Table S2C), while in *S. promiscuus* and *U. pinnatifida*, no false positives were identified.

For *S. promiscuus*, two *APT*-crRNAs were used simultaneously, causing insertions or deletions (Figure 2E). Most mutants had only one mutation near the crRNA target sites, whereas in two cases, approximately 1.5 kb between the two target sites were excised (Figure 2E). Additionally, one inversion occurred between the two target sites in one of the screened individuals (Figure 2E). In *L. digitata*, a single crRNA was used for each PEG transfection leading to small deletions (−1 to −43 bp) (Figure 2F).

Because *U. pinnatifida* is cultivated at 20°C and mesophilic Cas proteins such as SpCas9 may retain considerable activity at this temperature,^25^ we assayed the transformation efficiency of both Cas9 and LbCas12a RNPs employing two APT1 RNPs. All nucleases and crRNAs were shown to be functional (Figure 2G) with LbCas12a showing higher mutation efficiency (Table S2C) and yielding a wide range of genetic edits, including small deletions (Table S2D). Additionally, we noticed that *U. pinnatifida* exhibited higher 2-FA tolerance. Though 20 μM 2-FA concentration fully inhibits *Ectocarpus* gamete development and is lethal for *S. promiscuus* and *L. digitata*, untransformed *Undaria* could still develop in seawater supplemented with up to 20 μM 2-FA. To determine the optimal 2-FA concentration, we grew untransformed *Undaria* spores in different 2-FA concentrations (Figures S1C and S1D; Table S3) showing that 2-FA concentrations mentioned above caused severe developmental arrest (Figures S1C and S1D). Because transformants could be clearly distinguished by their ability to branch with 75 μM 2-FA, we employed this concentration for further transformant selection (Figures S1C and S1D; Table S3).

Overall, our results support that PEG-mediated LbCas12a-RNP complex transfection in gametes or meiospores can be easily scaled and applied to multiple brown algae species, including kelps of high economic and ecological importance.

## Discussion

Our method enabled efficient genome editing in four brown algal species using either gametes or meiospores. The introduced mutations were typically small deletions (∼50 bp) regardless of the species, while larger deletions or inversions could be induced by using multiple crRNAs targeting the same gene. The observed small deletions are consistent with patterns previously reported for Cas12a in other systems, including in the unicellular green alga *Chlamydomonas reinhardtii*.^26^^,^^27^ Since most brown algae produce parthenogenetic gametes, and even species without such gametes typically generate meiospores, our approach can be applicable to most brown algal species, provided that they produce sufficient numbers of wall-less zoids. In previous studies using biolistic delivery and microinjection in the model algae *Ectocarpus* sp 7, only an average of 1.2 and 0.9 *apt* mutants (2-FA-resistant individuals) were obtained per experiment, respectively,^12^ whereas our method can generate on average 30 individuals per trial. In some instances, even higher numbers have been isolated, which we speculate to be related to the quality and viability of the gametes isolated in each trial. Moreover, with biolistic transformation, fewer than half of the 2-FA-resistant individuals carried mutations in the *APT* gene, indicating a substantial proportion of false positives,^12^ which would require more intensive screening efforts than our method. In contrast, our method produced tens to thousands of 2-FA-resistant individuals per experiment across four different brown algal species, with false positives being absent in most experiments. By enabling precise, efficient, and accessible genetic manipulation, our approach opens the possibility to explore the molecular mechanisms underlying brown algal development, physiology, and evolution. Its high efficiency combined with high reproducibility and easy implementation will enable brown algae studies across a wide community of researchers. The improvement of the protocol therefore represents a major step forward for both fundamental research and biotechnological applications in this unique lineage of complex multicellular marine organisms.

### Limitations of the study

The effectiveness of our method may be limited in Fucales and Dictyotales since these groups lack gametes with parthenogenetic capacity.^28^ Additionally, Fucales do not produce meiospores and thus it is not possible to circumvent the lack of parthenogenic gametes while Dictyotales produce meiospores that are large and rich in cytoplasm,^28^^,^^29^ which could potentially interfere with transfection efficiency. These limitations could be circumvented by using brown algae protoplasts for PEG-mediated transfection, as originally reported in tobacco plants^30^ with the caveat that protoplast regeneration is time-consuming.^31^^,^^32^ Another possibility would be to transform male gametes and perform a cross immediately after transfection, which would then require further generations to isolate homozygous individuals. While microinjection is labor intensive and requires specialized equipment, introducing RNPs into vegetative cells or embryos via microinjection in Fucales and Dictyotales remains an alternative for these brown algal groups.

The CRISPR-Cas system has been widely used not only for gene KO but also for the targeted knockin (KI) of exogenous genes, although KI has not yet been achieved in brown algae. This study, however, did not address whether our PEG transfection could enable KI in brown algae. In the green alga *U. prolifera*, KI has been achieved by co-delivering single-stranded oligonucleotides (ssODNs) containing an exogenous gene sequence together with RNPs using PEG transfection.^33^ Considering that the KO efficiency in this study is comparable to that reported for PEG-mediated RNP delivery in *U. prolifera* (0.003%–0.16%),^11^ it is plausible that co-transfection of ssODNs could also enable KI in brown algae. This possibility remains to be investigated in future studies.

## Resource availability

### Lead contact

Requests for further information and resources should be directed to and will be fulfilled by the lead contact, Dr. Susana M. Coelho (susana.coelho@tuebingen.mpg.de).

### Materials availability

The algal culture strains will be made available through request to the lead contact. It may require a payment and/or a completed materials transfer agreement.

### Data and code availability

• All data are available in supplementary tables, and the genome data are available at https://phaeoexplorer.sb-roscoff.fr/. All data reported in this paper will be shared by the lead contact upon request.
• This paper does not report original code.
• Any additional information required to reanalyze the data reported in this paper is available from the lead contact upon request.

## Acknowledgments

We thank Dr. Kensuke Ichihara for valuable advice. We thank Agnes Henschen for technical support and Andrea Belkacemi and Dorothe Koch for assistance with algal cultures. This study was funded by the 10.13039/501100004189Max Planck Society, the 10.13039/501100000781European Research Council grant 864038 (S.M.C.), the Bettencourt Foundation (S.M.C.), and the Moore Foundation (S.M.C.). M.H. was supported by 10.13039/501100001691JSPS 10.13039/501100001691KAKENHI grant no. 23K19386, JSPS Overseas Research Fellowships, and Max Planck Partner Groups.

## Author contributions

C.M., M.H., and S.M.C. conceived and designed the experiments. C.M., V.B., M.H., and M.R. developed protocols. C.M., V.B., M.H., A.K., K.A.B., R.L., and M.R. performed the experiments. C.M., M.H., and S.M.C. wrote the manuscript with help of all authors. All authors provided critical feedback and helped shape the research, analysis, and manuscript writing.

## Declaration of interests

The authors declare no competing interests.

## STAR★Methods

### Key resources table

**Table:** 

REAGENT or RESOURCE	SOURCE	IDENTIFIER
**Chemicals, peptides, and recombinant proteins**		

Cas12 (Alt-R L.b. Cas12a [Cpf1])	IDT	Cat# 10007923
Alt-R™ S.p. HiFi Cas9 Nuclease V3, 500 μg	IDT	Cat# 1081061
Alt-R™ CRISPR-Cas9 tracrRNA, 5 nmol	IDT	Cat# 1072532
Polyethylene glycol 4000	Sigma	Cat# 8.07490
Polyethylene glycol 8000	Fisher BioReagents	Cat#10407773
Nuclease Free Duplex Buffer	IDT	Cat# 11-01-03-01
IDTE Buffer (pH 7.5)	IDT	Cat# 11-01-02-02
NEBuffer r3.1	New England Biolabs	Cat# B6003S
2-fluoroadenine (2-FA)	Sigma	Cat# 535087-1G
Nuclease-free water	Ambion	Cat# AM9932

**Critical commercial assays**		

Terra PCR Direct Polymerase Mix	Takara	Cat# 639271
KAPA3G Plant PCR Kit	RocheBiosystems	Cat# 3GPHSKB

**Experimental models: Organisms/strains**		

Strain Ec32 (*Ectocarpus* sp. 7 male)	N/A	N/A
Strain As6m (*Scytosiphon promiscuus* male)	N/A	N/A
*Laminaria digitata*	Field material (sporophytes) from Santec, France	N/A
Strain Un1f (*Undaria pinnatifida* female, Bizeux, St. Malo, France)	N/A	N/A
Strain Un1m (*Undaria pinnatifida* male, Bizeux, St. Malo, France)	N/A	N/A

**Oligonucleotides**		

Oligonucleotides used in the paper	This paper	see Table S1.

**Software and algorithms**		

DIAMOND	Buchfink et al.^34^	https://doi.org/10.1038/s41592-021-01101-x
CRISPOR	Concordet and Haeussler^35^	https://doi.org/10.1093/nar/gky354

### Experimental model and study participant details

#### Culture strains and field materials used in this study

All culture strains (see details in key resources table) are maintained in Department of algal development and evolution, Max Planck Institute for Biology, Tübingen. Sporophytes of L. *digitata* were collected at Santec, France on 8 December 2022, and transported to the lab. Note that we used male strains but these organisms have almost no sexual dimorphism and no difference is expected.

### Method details

#### *Ectocarpus* sp.7 PEG-mediated RNP transfection

*Ectocarpus* sp.7 gene sequences were retrieved from v3 reference genome^36^ and CRISPOR^35^ used to design LbCas12a crRNAs with limited off-targets and highest efficiency scores. Male gametophytes (strain Ec32) were cultured in plastic Petri dishes (150 × 15 mm) (10 individuals per Petri dish) and sterile natural sea water (NSW) enriched with half-strength Provasoli medium (Provasoli enriched sea water: PES) at 14°C in neutral day (12 h: 12 h, light:dark) conditions with LED lighting of 20 μmol m^−2^ s^−1^ photon flux density. Male gametophytes were isolated from individual unilocular sporangia, which in turn were isolated from Ec32 mature partheno-sporophytes (3-4-week old), as previously described.^37^ Mature gametophytes (displaying clear accumulation of mature plurilocular sporangia) are observed usually after 3–4 weeks after culture preparation and clearly visible under a light stereoscope.^37^^,^^38^ Gametophytes grown on 50 Petri dishes (500 gametophytes) were collected under a laminar flow hood on a sieve with a 50 μm mesh and rinsed with NSW at 14°C to remove small filaments and already released gametes. The biomass was concentrated and equally distributed in small Petri dishes (60 × 15mm) up to 100 gametophytes per Petri dish and the excess of water was removed with a 200 μL pipet. To maintain high humidity levels a few (typically 4–8) drops of NSW were added on the edge of the Petri dish. The dishes were sealed with parafilm, wrapped in aluminum foil to keep darkness conditions and transferred for 3 h to 14°C. Gamete release was induced by adding 5 mL of 4°C NSW and incubating 5 min at room temperature under 40 μmol m^−2^ s^−1^ photon flux density. Gametophytes were incubated further 30 min at 14°C with LED lighting of 40 μmol m^−2^ s^−1^ photon flux density to allow for further gamete release. The gametes were then separated from gametophytic tissue by filtering through a 10 μm cell strainer into a 50 mL conical plastic tube and concentrated by centrifuging in a swing-out rotor (4600 rpm) for 5 min at Room Temperature (RT). The majority of the supernatant was removed by pipetting and 200–300 μL of NSW were left to resuspend the gametes. The gamete suspension was then diluted 1:10 and stained with 1 μL of lugol solution to count in a haemocytometer. The gametes were then diluted to 1X10^4^/μL in NSW. For PEG-mediated transfection, in a laminar flow hood, 20 μL of RNP complex mixture (or 20 μL mock control with IDT buffer) were pipetted into a plastic Petri dish (90 × 20mm) and 100 μL of the gamete suspension (10^6^ gametes) were gently mixed by pipetting. This gamete/RNP solution was then mixed with 120 μL of 40% w/v PEG-8000 (Fisher Scientific, Schwerte, Germany) or PEG-4000 (Sigma, Darmstadt, Germany) solution, filtered through a 0.22 μm filter by pipetting up and down. PEG pipetting and mixing was carried out vigorously with a wide bore 200 μL filter tip to ensure proper homogenization. A small degree of bubbling is expected and did not, in our hands, influence transformation efficiency. The gametes were then incubated at RT in darkness for 30 min. The 20 μL of the RNP complex was prepared as follows: 1.2 μL of crRNA (100 μM, IDT, Leuven, Belgium), 2.8 μL of IDTE Buffer (IDT), 8 μL of LbCas12a (Alt-R L.b. Cas12a [Cpf1], 15.6 μM, diluted from a 67 μM stock, IDT), 8 μL of 2.5X NEB Buffer 3.1 (New England Biolabs, Ipswich, MA, USA). For transfections involving multiple RNP complexes, each complex was prepared separately, then combined in the Petri dish, the amount of PEG solution was then adjusted accordingly to maintain a 1:1 ratio (Gamete/RNP:PEG). Following transfection, 20 mL of half-strength PES was added to each plate to stop transfection and enable gamete germination. Dishes were incubated overnight in the dark at 22°C (typically 16 h), then transferred the next morning to 14°C in neutral day conditions (12 h: 12 h, light:dark). 48h after transformation, 2-fluoroadenine (2-FA, Sigma) was added to the Petri dishes to a final concentration of 20 μM, and samples were incubated in neutral day conditions (12 h: 12 h, light:dark) with LED lighting of 20 μmol m^−2^ s^−1^ photon flux density until the isolation of 2-FA resistant algae and therefore bearing putative mutations in *APT* and the gene of interest.

Six to eight weeks after the transfection, the number of growing germlings in 2-FA supplemented culture medium (putative *apt* mutants) were counted, and some of them were carefully isolated to 2-FA free half-strength PES using forceps to generate enough biomass for genotyping and validation. Genotyping PCR was performed using the Terra PCR Direct Polymerase Mix (Takara) or the Kapa G3 Plant PCR kit (RocheBiosystems) with 2 μL of the following suspension: small fragments of algal tissue (approximately 1 mm^2^) ground in 60 μL of Nuclease free Water (Ambion). Sanger sequencing was performed by Azenta. Details of cRNA and primers are provided in Table S1.

#### *Scytosiphon promiscuus* PEG transfection

The *APT* gene of *S. promiscuus* was identified by aligning protein sequence of the *Ectocarpus* sp.*7 APT* gene against the *S. promiscuus* proteome^39^ using DIAMOND.^34^ A single gene (gene code: mRNA_S-promiscuus_M_contig7.17323.1) on chromosome 28 was identified as *APT* gene of *S. promiscuus*.

Male gametophytes (strain As6m) were cultivated in plastic Petri dishes (90 × 20 mm) and sterile NSW enriched with full strength PESI medium^40^ at 10°C in long day (16h:8h, light:dark) conditions with LED lighting of 20 μmol m^−2^s^−1^ photon flux density. Medium was renewed every week until gamete collection. Mature gametophytes release gametes immediately after the medium renewal and thus cultures were closely inspected after each media change. Because *S. promiscuus* gametes have negative phototaxis, freshly released gametes accumulate on the opposite side of a light source in a Petri dish. The accumulated gametes were collected and diluted to 0.4–10 million per 1 mL with NSW. For PEG transfection, 100 μL of the gamete suspension was gently mixed with 8 μL of RNP complex and 108 μL of 40% w/v PEG-4000 solution in a plastic Petri dish (90 × 20 mm), and then, incubated at room temperature in dark for 30 min. To prepare 8 μL of the RNP complex, two different crRNAs were mixed as following:1 μL of crRNA1 (100 μM), 1 μL of crRNA2 (100 μM), 1.5 μL of LbCas12a (67 μM), 3.2 μL of 2.5X NEB Buffer 3.1, and 1.3 μL of water. After the transfection, 50 mL of full-strength PESI was added to the Petri dishes and incubated in a 22°C dark condition for 48 h. Then, 2-FA was added to the Petri dishes (final 2-FA concentration of 20 μM), and the samples were incubated in a 14°C neutral day condition until the isolation of putative *APT* mutants. One month after the transfection, the number of growing germlings in 2-FA supplemented culture medium (putative *apt* mutants) were counted, and eight of them were isolated using glass Pasteur pipets, before being genotyped to validate the mutations. Genotyping was performed as in *Ectocarpus*. Details of crRNA and primers are provided in Table S1.

#### *Laminaria digitata* PEG transfection

The *APT* gene of *Laminaria. digitata* was identified by aligning *Ectocarpus* sp. 7 *APT* gene against *L. digitata* proteome^39^ as described above. The *APT* gene of *Laminaria* (gene codes: mRNA_L-digitata_M_contig27133.1.1 and mRNA_L-digitata_M_contig6833.2.1) was divided into two contigs (contig27133 and contig6833) because of the fragmented reference genome assembly.

Sporophytes collected from the field were washed with sterile NSW and pat-dried to remove water. The sori were excised from the sporophytes and transferred to the laboratory in a chilled transport box. To induce meiospore release, the sori were placed in fresh sterile NSW at room temperature one day after the sampling. However, this did not result in immediate meiospore release. The sori were therefore incubated in sterile NSW in 14°C neutral day conditions until meiospores were released up to two days. Meiospores were collected from three independent sporophyte individuals. Meiospore release resulted in a change in water color toward brown tones. This brown seawater was transferred to 50 mL tubes and centrifuged at 4000g for 1 min. After the centrifugation, the supernatant was removed and fresh sterile seawater was added to the pellet of meiospores and the density of meiospores was adjusted to 0.54–7.7 million per 1 mL. PEG transfection was performed as in *S. promiscuus*, except that each cRNA was used independently. The RNP complex were prepared as following: 1 μL of crRNA (100 μM), 0.75 μL of LbCas12a (67 μM), 3.2 μL of 2.5X NEB Buffer 3.1, and 3.05 μL of water. After the transfection, 50 mL of full-strength PESI was added to the Petri dishes and incubated in a 22°C dark condition for 48 h. Then, 2-FA was added to the Petri dishes to reach a concentration of 20 μM and incubated in a 14°C neutral day condition (green light) until the isolation of putative *apt* mutants. Some of these putative mutants were isolated and genotyped to confirm the mutations. Genotyping was performed as in *Ectocarpus*. Details of crRNA and primers are provided in Table S1.

#### *Undaria pinnatifida* PEG transfection

The *APT* gene of *Undaria pinnatifida* was identified by aligning *Ectocarpus* sp. 7 *APT* gene against the *U. pinnatifida* proteome,^41^ as described above. The *APT* gene of *Undaria* (UNPIN0032CG0760) is located on contig LG22.

Mature sporophytes of *U. pinnatifida* were obtained by crossing the gametophyte strains Un1f and Un1m, isolated from a single sporophyte (Bizeux, St. Malo, France) cultured in sterile NSW enriched with half-strength PES under controlled laboratory conditions (16°C–20°C, 16:8 h light:dark) with LED lighting at 10 μmol m^−2^ s^−1^ photon flux density. Spore release was induced by transferring sporophyll tissue to fresh NSW and incubating at room temperature with gentle shaking for 20 min. The spore suspension was then collected and its concentration was measured using a haemocytometer. A suspension of approximately 7.5×10^5^ spores per 100 μL was used for transfection.

For PEG transfection, 100 μL of the spore suspension was gently mixed with 20 μL of RNP complex and 120 μL of 40% w/v PEG-8000 (Fisher Scientific, Schwerte, Germany), filtered through a 0.22 μm syringe filter, in a sterile plastic Petri dish (90 × 20 mm), and then incubated at room temperature in the dark for 30 min 20 μL of RNP complex was prepared as follows: for Cas9, 4 μL of crRNA:tracrRNA duplex (100 μM each, IDT, Leuven, Belgium), annealed in IDT Duplex Buffer, 8 μL of Cas9 enzyme (15.6 μM, IDT), and 8 μL of 2.5X NEB 3.1 Buffer (New England Biolabs, Ipswich, MA, USA); for LbCas12a, 4 μL of crRNA (100 μM, IDT Leuven, Belgium), 8 μL LbCas12a (15.6 μM, diluted from a 67 μM stock), and 8 μL of 2.5X NEB 3.1 Buffer. For dual RNP transfections, each complex was prepared separately and combined in the dish prior to PEG addition.

Following transfection, 20 mL of sterile NSW supplemented with half-strength PES was added to each dish. The plates were incubated overnight at room temperature in the dark and then transferred the next morning to normal culture conditions at 20°C with a 16:8 light:dark cycle.

Forty-eight hours after transformation, 2-fluoroadenine (2-FA, Sigma) was added to each plate at a final concentration of 75 μM. The final concentration of 75 μM 2-FA was selected based on a sensitivity assay on *U. pinnatifida* spores (Figure S1C; Table S3). Samples were incubated under standard growth conditions with LED lighting of 10–20 μmol m^−2^ s^−1^ photon flux density for one week. Branching 2-FA-resistant gametophytes were isolated and grown individually for further genotyping to validate *APT*-targeted mutations using Sanger sequencing as in *Ectocarpus*. Details of crRNA and *APT* primers are provided in Table S1.

### Quantification and statistical analysis

#### Statistical tests regarding the protocol optimization for ectocarpus

In the dot plot in Figures 1C, 1D, S1A, and S1B, dots depict independent trials (Table S2), and the horizontal and vertical lines represent the mean and standard deviation, respectively. Significance was determined using an Exact Wilcoxon rank-sum test, and *p* < 0.05 were considered significant. Statistical analysis was performed using R software v. 4.2.2.

#### Transfection efficiency and false positive rate of PEG transfection

Transfection efficiency (expected mutation efficiency in Table S2C) was calculated by dividing the number of germlings that survived on 2FA medium (the number of branched germlings in *Undaria*) by the number of zoids used for transfection. The false positive rate was determined by dividing the number of 2FA-resistant individuals in which no mutation was detected at the APT locus by the total number of genotyped 2FA-resistant individuals (possible *APT* mutants). The corrected transfection efficiency (corrected expected mutation efficiency considering the false positive rate in Table S2C) was obtained by multiplying the transfection efficiency by (1 − false positive rate).

## Supplemental information

Document S1. Figure S1

Table S1. List of crRNAs and primers used in the present study, related to Figures 1 and 2 and STAR Methods

Table S2. Results summary of PEG experiments, related to Figures 1 and 2 and STAR Methods (A) Optimization of PEG condition for *Ectocarpus*. (B) Result summary of *Ectocarpus IMM* mutagenesis. (C) Results summary of *APT* mutagenesis in *Scytosiphon*, *Laminaria*, and *Undaria*. (D) Summary of *Undaria APT* mutants

Table S3. Sensitivity of *Undaria* to 2-fluoroadenine (2-FA), related to STAR methods

Document S2. Article plus supplemental information
