# Supplementary material for: Efficient CRISPR-Cas genome editing in brown algae
Source: Cell Rep Methods. 2025 Dec 30;6(1):101273. doi: 10.1016/j.crmeth.2025.101273 (PMC12853178; doi:10.1016/j.crmeth.2025.101273)
Supplement: Document S1. Figure S1 [file mmc1.pdf]

Cell Reports Methods, Volume *6*

Supplemental information

Efficient CRISPR-Cas genome editing in brown algae

Cláudia Martinho, Masakazu Hoshino, Morgane Raphalen, Viktoriia Bukhanets, Anagha Kerur, Kenny A. Bogaert, Rémy Luthringer, and Susana M. Coelho

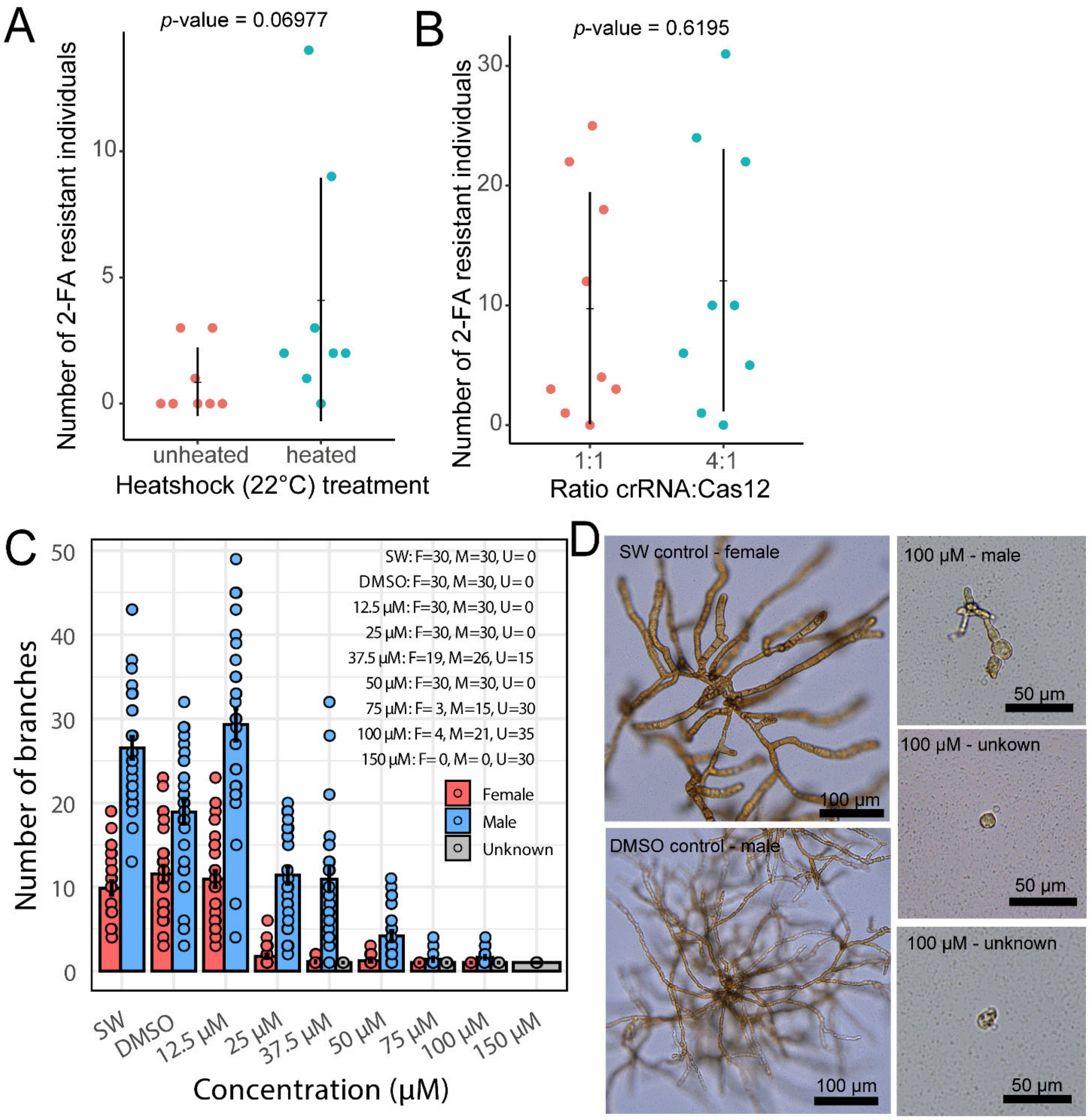

Figure S1. **Protocol optimization for *Ectocarpus* and *Undaria*, related to Figure 1, 2 and STAR Methods. (A)** Scatter plot showing the effect of the heat shock at 22ºC on the number of 2-FA resistant individuals in *Ectocarpus*. The horizontal and vertical lines in the scatter plot represent the mean and standard deviation, respectively. No significant effect was detected (exact Wilcoxon rank sum test, $p = 0.06977$). (**B)** Scatter plot showing the effect of the crRNA:LbCas12a ratio (1:1 vs. 4:1) on the number of 2-FA resistant individuals in *Ectocarpus*. No significant effect was detected (exact Wilcoxon rank sum test, $p = 0.6195$). **(C)** Quantification of *Undaria* gametophyte branching across increasing 2-FA concentrations 14 days after 2-FA addition. Each dot depicts the number of branches per individual, and bars indicate mean ± SE for each treatment and sex (female (F) or male (M)). Sex was inferred from the filament diameter, while very small or developmentally arrested individuals that could not be phenotyped were classified as "unknown" (U). Higher 2-FA concentrations caused strong developmental arrest and reduced branching, but not complete lethality among the seeded meiospores. **(D)** Representative *Undaria* individuals showing a control female (SW control, top left) and a control male (DMSO control, bottom left), as well as individuals exposed to 100 µM 2-FA displaying arrested or slowly developing morphologies classified as male or unknown (right).
